# Low dose of morphine to relieve dyspnea in acute respiratory failure: the OpiDys double-blind randomized controlled trial

**DOI:** 10.1186/s12931-024-02867-2

**Published:** 2024-07-16

**Authors:** Robin Deleris, Côme Bureau, Saïd Lebbah, Maxens Decavèle, Martin Dres, Julien Mayaux, Thomas Similowski, Agnès Dechartres, Alexandre Demoule

**Affiliations:** 1 AP-HP, Groupe Hospitalier Universitaire APHP-Sorbonne Université, site Pitié-Salpêtrière, Service de Médecine Intensive et Réanimation (Département R3S), F-75013 Paris, France; 2 Sorbonne Université, INSERM, UMRS1158 Neurophysiologie Respiratoire Expérimentale et Clinique, F-75005 Paris, France; 3 Département Biostatistique Santé Publique et Information Médicale, Centre de Pharmacoépidémiologie (Cephepi), Groupe Hospitalier Universitaire APHP-Sorbonne Université, site Pitié-Salpêtrière, Paris, France; 4 Sorbonne Université, INSERM, Institut Pierre Louis d’Epidémiologie et de Santé Publique, CIC-1421, F75013 Paris, France; 5 AP-HP, Groupe Hospitalier Universitaire APHP-Sorbonne Université, site Pitié-Salpêtrière, Département R3S, F-75013 Paris, France

**Keywords:** Dyspnea, Morphine, Intensive care unit, Acute respiratory failure, Intubation, Trial

## Abstract

**Background:**

Morphine relieves dyspnea in various clinical circumstances. Whether or not this applies to patients admitted to intensive care units (ICUs) for acute respiratory failure (ARF) is unknown. We evaluated the efficacy and safety of low-dose morphine on dyspnea in patients admitted to the ICU for ARF.

**Methods:**

In this single-center, double-blind, phase 2, randomized, controlled trial, we assigned non-intubated adults admitted to the ICU for ARF with severe dyspnea, defined by a visual analog scale for dyspnea (dyspnea-VAS) from zero (no dyspnea) to 100 mm (worst imaginable dyspnea) ≥40 mm, to receive a low dose of Morphine Hydrochloride (intravenous titration followed by subcutaneous relay) or Placebo. All patients received standard therapy, including etiological treatment and non-invasive respiratory support.

**Results:**

Twenty-two patients were randomized, 11 in each group. The average dyspnea (median [interquartile range]) over 24 hours did not significantly differ between the two groups (40 [25 – 43] mm in the Morphine group vs. 40 [36 – 49] mm in the Placebo group, *p*=0.411). Dyspnea-VAS was lower in the Morphine group than in the Placebo group at the end of intravenous titration (30 [11 – 30] vs. 35 [30 – 44], *p*=0.044) and four hours later (18 [10 – 29] vs. 50 [30 – 60], *p*=0.043). The cumulative probability of intubation was higher in the Morphine group than in the Placebo group (*p*=0.046)

**Conclusion:**

In this phase 2 pilot trial, morphine did not improve 24-hour average dyspnea in adult patients with ARF, even though it had a statistically significant immediate effect. Of concern, Morphine use was associated with a higher intubation rate.

**Trial registration:**

The protocol was declared on the ClinicalTrial.gov database (no. NCT04358133) and was published in September 2022.

**Supplementary Information:**

The online version contains supplementary material available at 10.1186/s12931-024-02867-2.

## Introduction

Dyspnea is one of the most distressing experiences a human being can endure [1]. Approximately half of patients admitted to the intensive care unit (ICU) for acute respiratory failure (ARF) report moderate to severe dyspnea [2]. Average dyspnea intensity in this population is 40 mm on a visual analog scale (VAS) ranging from zero (no dyspnea) to 100 mm (worst imaginable dyspnea) [2, 3]. Patients undergoing non-invasive ventilation report dyspnea as one of the worst experiences of their ICU stay [4]. In this population, there is a strong association between dyspnea and anxiety [5]. Finally, dyspnea is associated with a higher intubation rate [4, 6] and a higher mortality [6]. It should be noted that in intubated patients, dyspnea is associated with an increased prevalence of post-traumatic stress disorder [5]. For all these reasons, controlling dyspnea in ARF patients is a major goal of care [7].

Unfortunately, dyspnea can persist in spite of the optimal treatment of the condition causing ARF, oxygen supplementation and non-invasive ventilatory support or the correction of metabolic abnormalities [8]. Opioids, well known to relieve dyspnea [9], could help in controlling dyspnea in ARF patients [10]. The fear of overdose with respiratory depression has historically been the main obstacle to the widespread use of morphine for the relief of dyspnea. However, several meta-analyses have shown the benefit of morphine on long-term persistent dyspnea, but also its safety in patients with end-stage onco-hematological disease, chronic obstructive pulmonary disease or advanced heart failure [9–25]. In addition, recent guidelines from the American Thoracic Society advocate oral or parenteral administration of opioids for persistent dyspnea [26].

The objective of this trial was to determine whether the administration of low-dose titrated morphine, compared to placebo, in patients admitted to the ICU for ARF with moderate to severe dyspnea decrease dyspnea without increasing adverse events.

## Methods

### Trial design

This is a single-center phase 2 double-blind randomized controlled trial conducted in a 22-bed medical ICU within La Pitié-Salpêtrière University Hospital in Paris, France. The trial was approved by the Institutional Review Board (South Mediterranean III Comité de Protection des Personnes on December 5, 2019, no. 19.10.24.60836). All patients or relatives provided written informed consent. The protocol was declared on the ClinicalTrial.gov database (no. NCT04358133) and was published in September 2022 [27].

### Participants

Eligibility criteria were patients on standard oxygen, high-flow oxygen or non-invasive ventilation who fulfilled all the following criteria, 1) admitted to the ICU for an ARF defined as a respiratory rate >24/min or signs of respiratory distress such as labored breathing or paradoxical inspiration, or SpO2 <90% in ambient air; 2) with dyspnea ≥40 mm on a VAS for dyspnea (dyspnea-VAS) from zero (no dyspnea) to 100 mm (worst imaginable dyspnea) despite the department's usual measures: analgesic and anxiolytic treatment, reassurance, etiological treatment of ARF, and non-invasive respiratory support; 3) with age between 18 and 75 years; 4) Richmond agitation and sedation scale (RASS) between 0 and +2; 5) who presented no confusion, as defined by the Confusion Assessment Method for ICU (CAM-ICU) [28]; 6) who provided informed consent or for whom consent could be obtained from a relative or through emergency consent procedure.

Non-inclusion criteria were intubated and tracheotomized patients or patients whose intubation was planned upon admission; patients unable to communicate verbally and self-report dyspnea on a VAS (hearing or visual impairment, insufficient command of French, previous known psychiatric or cognitive disorders; moribund patients; contraindication to opioids (known hypersensitivity to opioids, creatinine clearance <30 ml/min, severe hepatocellular insufficiency defined by factor V <50%); pregnant or breastfeeding woman; opioid use within the 24 hours before inclusion; protected adult; not affiliated to the French public health insurance: previous inclusion in this trial; exclusion period due to inclusion in another clinical trial.

### Randomization

After informed consent had been obtained, participants were included in the study and randomly assigned in a 1:1 ratio to the intervention or control group using a computer sequence with random permuted blocks. Randomization was performed on the electronic case report form (eCRF) (Cleanweb, Télémédecine Technologies, Boulogne-Billancourt, France). Sequentially numbered containers of identical appearance prepared by the pharmacy and containing morphine or placebo were stored in the ICU. The container with the smallest serial number available in the department's stock was assigned to the newly included patient.

### Intervention

All management decisions other than the administration of morphine were made by the managing physician according to the department's usual practices.

**Fig. 1:**
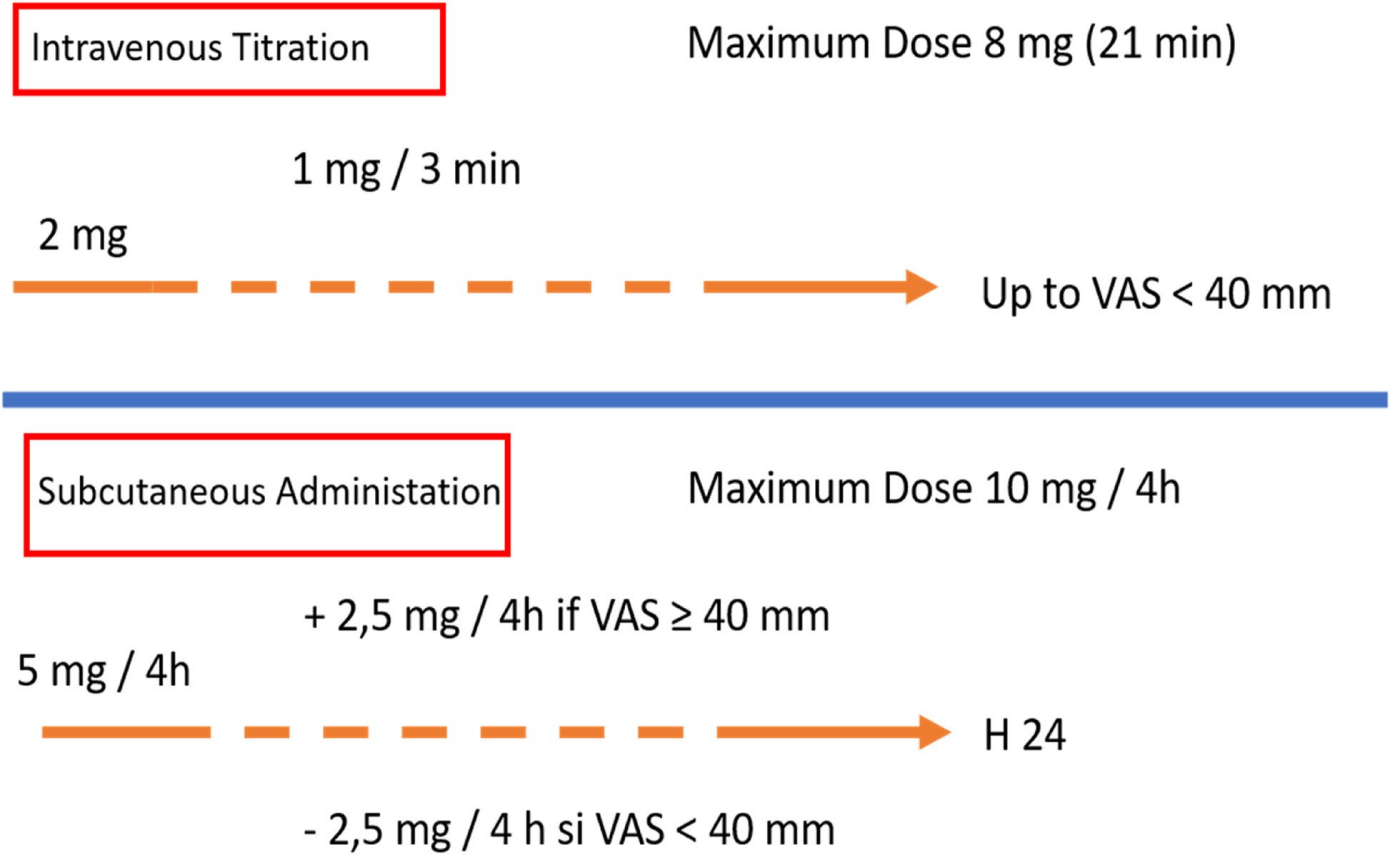
Procedure for administering morphine hydrochloride 1 mg/mL or placebo. The experimental group received an intravenous titration of morphine hydrochloride at a concentration of 1 mg per ml of NaCl 0.9%. The titration consisted of an initial bolus of 2 ml, followed by a 1 ml bolus every 3 minutes until dyspnea-VAS was <40 mm, with a maximum safety dose of 8 mg. Once the target (either dyspnea-VAS <40 mm or safety dose of 8 ml) was reached, morphine hydrochloride (1 mg per ml) was administered subcutaneously. A first dose of 5 ml was administered immediately after intravenous titration and then every 4 hours for 24 hours. At each 4-hour time point, if dyspnea-VAS was ≥40 mm, the dose of morphine was increased from the previous one by increments of 2.5 ml, without exceeding the maximum dose of 10 ml every 4 hours. If Dyspnea-VAS was <40 mm, the dose of morphine administered every 4 hours was reduced by 2.5 ml. The control group received NaCl 0.9%, which was administered according to the same protocol as the experimental arm

The experimental group received an intravenous titration of morphine hydrochloride at a concentration of 1 mg per ml of NaCl 0.9%. The titration consisted of an initial bolus of 2 ml (2 mg), followed by a 1 ml (1 mg) bolus every 3 minutes until dyspnea-VAS was <40 mm, with a maximum safety dose of 8 ml (8 mg). Once the target (either dyspnea-VAS <40 mm or safety dose of 8 mg) was reached, morphine hydrochloride (1 mg per ml) was administered subcutaneously. A first dose of 5 ml (5 mg) was administered immediately after the intravenous titration and then every 4 hours for 24 hours. At each 4-hour time point, if dyspnea-VAS was ≥40 mm, the dose of morphine was increased from the previous one by increments of 2.5 ml, without exceeding the maximum dose of 10 ml (10 mg) every 4 hours. If Dyspnea-VAS was <40 mm, the dose of morphine administered every 4 hours was reduced by 2.5 ml (2.5 mg) (Fig. 1).

The control group received NaCl 0.9%, which was administered according to the same protocol as the experimental arm (Fig. 1).

### Outcomes

Primary outcome was the average of the dyspnea ratings gathered every 4 hours over the 24 hours following inclusion or until intubation. The following secondary outcomes were measured over the first 24 hours following randomization: intensity of dyspnea-VAS at the end of the intravenous titration and every 4 hours; average anxiety-VAS, respiratory rate and Glasgow coma scale measured every 4 hours; incidence of moderate-to-severe dyspnea and anxiety, defined by a VAS ≥40 mm; intubation rate; incidence of Glasgow coma scale ≤12; incidence of delirium defined by the CAM-ICU, duration and quality of sleep during the first night as assessed by the patients themselves (informally) at the end of the first night by a VAS (from 0, worst to 100 mm, best); proportion of patients requiring the transition from one oxygenation technique to another; number of non-invasive ventilation sessions; total duration of standard oxygen, non-invasive ventilation and high-flow nasal oxygen; tolerance of standard oxygen, high-flow nasal and non-invasive ventilation (VAS from 0, worst to 100 mm).

Constipation, nausea and severity of dry eye, dry nose and feeling of gastric distension were evaluated at the end of the 24-hour study period (VAS from 0, worst to 100 mm, best). Nurses’ adherence to and satisfaction with the protocol were evaluated at the end of the 24-hour study period (VAS from 0, worst to 100 mm, best).

The following adverse events considered medically significant occurring within the first 48 hours were collected: intubation; nausea ≥grade 3; constipation ≥grade 4; bradypnea <12 cycles per minute; coma defined by a Glasgow coma scale ≤9, pruritus grade ≥4; [29] worsening of respiratory condition requiring intubation.

### Statistical analysis

Based on previous data, we hypothesized that mean dyspnea-VAS over the first 24 hours would be 37 mm in the control arm with a standard deviation of 26 mm [2, 3]. We hypothesized that mean dyspnea-VAS over the first 24 hours would be 12 mm in the experimental arm, which makes a difference of 25 mm, which is which is more than twice the minimally clinical important difference (10 mm) for dyspnea-VAS in other clinical contexts [30]. Therefore, with a power of 80% and a one-sided alpha risk of 10%, we calculated that 22 patients should be recruited (11 per group). The choice of a one-sided alpha risk of 10% is justified by the fact that we did not want to miss a potential signal of an effect of morphine on dyspnea in this phase 2 pilot study.

The analysis used the intent-to-treat approach, ie, all patients were analyzed in the group allocated by randomization, with no exclusion after randomization except exclusions for withdrawn consent according to the French regulation. Categorical variables were described as frequency and percentage and quantitative variables were described as median and interquartile range.

For the primary outcome, the comparison between the two treatment groups of the average dyspnea during the first 24 hours was performed by a Wilcoxon’s rank-sum test, taking a one-sided alpha risk of 10% to limit the risk of missing a difference.

For secondary outcomes, quantitative variables were compared between the two arms with a Wilcoxon’s rank-sum test. Categorical variables were compared between the two arms with a Fisher’s exact test. Cumulative probability of intubation was compared with the log rank test. All analyses were carried out with a unilateral alpha risk of 10%, using R software Version 4.1.1.

## Results

### Patients characteristics and intervention

**Fig. 2:**
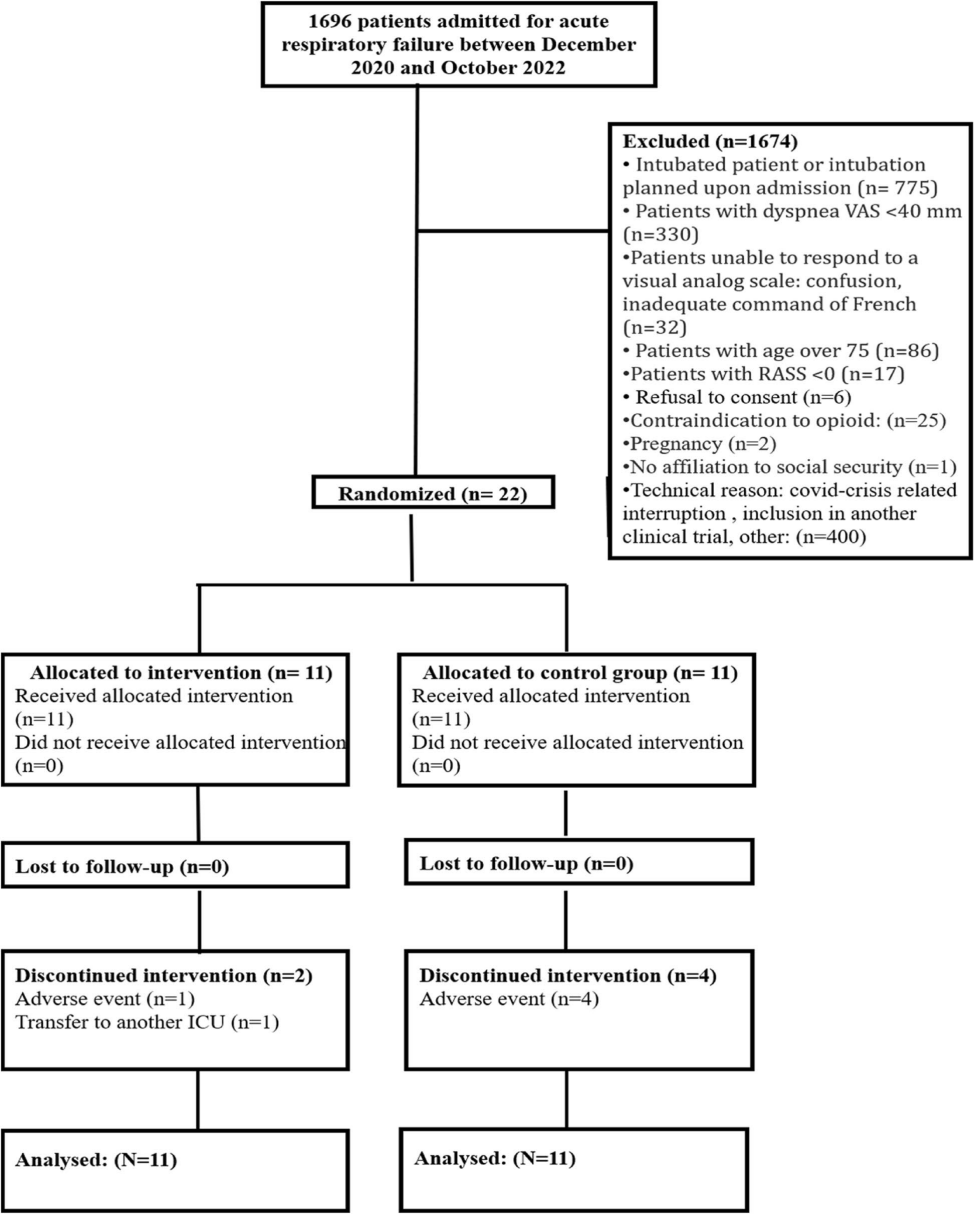
Study flowchart

From December 16, 2020 to October 7, 2022, 1696 patients were admitted for ARF, and 22 patients were randomized: 11 in the Placebo group and 11 in the Morphine group. Because of the particular feature of dyspnea in COVID-19 patients, the study was interrupted during the pandemic. Figure 2 shows the study flow chart and reasons for not including patients. Baseline characteristics were evenly distributed between the two groups (Table 1).

**Table 1:** Patient characteristics at baseline

	**All patients** **(*n*=22)**	**Placebo** **(*n*=11)**	**Morphine** **(*n*=11)**
Age, *years, median (IQR)*	65 [55 – 69]	68 [64 – 70]	57 [51 – 66]
Male gender, *n (%)*	18 (82)	10 (91)	8 (73)
BMI, *kg.m*^*-2*^*, median (IQR)*	27 [23 – 31]	26 [23 – 30]	28 [23 – 31]
Chronic respiratory disease, *n (%)*	14 (64)	6 (55)	8 (73)
Chronic heart disease, *n (%)*	11 (50)	8 (73)	3 (27)
Chronic neurologic disease, *n (%)*	1 (5)	0 (0)	1 (9)
**Chronic medication**			
Antalgic, *n (%)*	1 (5)	1 (9)	0 (0)
Anxiolytic, *n (%)*	2 (9)	0 (0)	2 (18)
**Cause of ARF**			
Acute-on-chronic ARF, *n (%)*	6 (27)	2 (18)	4 (36)
Acute cardiogenic pulmonary edema, *n (%)*	2 (9)	2 (18)	0 (0)
De novo ARF, *n (%)*	11 (50)	5 (45)	6 (55)
Other, *n (%)*	3 (14)	2 (18)	1 (9)
SAPS 2 on ICU admission,* median (IQR)*	29 [22 – 38]	29 [26 – 37]	32 [21 – 41]
**On the day of enrolment in the study**			
Anxiety-VAS, *median (IQR)*	56 [8 – 69]	51 [15 – 62]	60 [22 – 89]
Dyspnea-VAS, *median (IQR)*	70 [59 – 80]	70 [51 – 74]	70 [60 – 80]
Respiratory rate, *per min, median (IQR)*	26 [23 – 31]	25 [22 – 29]	29 [23 – 32]
SpO_2_ in ambient air, *%, median (IQR)*	84 [80 – 88]	82 [80 – 89]	85 [80 – 88]
Labored breathing, yes, *n (%)*	12 (55)	6 (55)	6 (55)
Systolic BP, *mmHg, median (IQR)*	126 [111 – 145]	119 [110 – 137]	131 [123 – 144]
Heart rate, *per min, median (IQR)*	94 [86 – 103]	87 [85 – 100]	95 [90 – 104]
Creatinine clearance, *ml/min, median (IQR)*	92 [72 – 106]	84 [62 – 106]	95 [84 – 100]
**Arterial blood gases**			
PaO_2_/FiO_2_, *mmHg, median (IQR)*^a^	157 [116 – 274]	156 [122 – 309]	158 [107 – 247]
PaCO_2_, *mmHg, median (IQR)*	38 [32 – 48]	40 [34 – 50]	36 [31 – 43]
pH, *mmHg, median (IQR)*	7.43 [7.34 – 7.47]	7.43 [7.35 – 7.46]	7.43 [7.31 – 7.48]
HCO^3^, *mmol.L-1, median (IQR)*	27 [24 – 30]	28 [25 – 32]	27 [22 – 27]
**Ventilator settings**			
Standard oxygen, *n (%)*	14 (64)	7 (64)	7 (64)
Non-invasive ventilation, *n (%)*	3 (14)	1 (9)	2 (18)
High-flow oxygen therapy, *n (%)*	5 (23)	3 (27)	2 (18)

Quantitative variables are expressed as median (interquartile range [IQR]) and qualitative variables are expressed as frequency (percentage)

*BMI* body mass index, *ICU* intensive care unit, *SAPS 2* Simplified Acute Physiology Score, *ARF* acute respiratory failure, *VAS* visual analog scale, *BP* blood pressure, PaO_2_/FiO_2_ ratio of arterial oxygen tension to inspired oxygen fraction

^a^In patients on standard oxygen, FiO_2_ was measured as follows: (oxygen flow in liters per minute) x 0.3 + 0.21

Dyspnea-VAS upon inclusion was severe in both groups (70 [51 – 74] mm in the Placebo group and 70 [60 – 80] mm in the Morphine group). During titration, patients in the Morphine group received 3 [2–6] mL of morphine hydrochloride 1 mg/ml vs. 5 [4–7] ml in the Placebo group. The proportion of patients who reached a dyspnea-VAS <40 mm at the end of the titration was 91% (*n*=10) in the Morphine group vs. 73% (*n*=8) in the Placebo group. Time to reach a dyspnea-VAS <40 mm or the maximum intravenous dose of 8 ml was 6 [3–18] min in the Morphine group and 13 [9–18] min in the Placebo group (*p*=0.431). Over the 24 hours following titration, patients in the Morphine group received 8 [8–21] ml of morphine hydrochloride 1 mg/ml vs. 28 [15–35] ml in the Placebo group.

Intervention was discontinued in four patients of the Morphine group because of respiratory failure requiring intubation and in one patient of the Placebo group because of a transfer to another ICU. In the Morphine group, two of the four intubations occurred during the intravenous administration phase, while the two others occurred during the subcutaneous administration phase.

During the follow-up, 3 patients (27%) in the Morphine group and 2 patients (18%) in the Placebo group received anxiolytics (*p*>0.999), 1 patients (9%) in the Morphine group and 4 patients (36%) in the Placebo group received non-opioid analgesics (*p*=0.31), and 3 patients (27%) in the Morphine group and 6 patients (55%) in the Placebo group received either anxiolytics, non-opioid analgesics or both (*p*=0.39).

### Primary outcome

Average dyspnea-VAS over the 24 hours following inclusion was 40 [25 – 43] mm in the Morphine group and 40 [36 – 49] mm in the Placebo group (*p*=0.411) (Table 2).

**Table 2:** Primary and secondary outcomes over the 24 hours following randomization

**Endpoints**	**All patients** **(*n*=22)**	**Placebo** **(*n*=11)**	**Morphine** **(*n*=11)**	***P* value**
***Primary endpoint***				
Average dyspnea-VAS over the first 24 hours, *median (IQR)*	40 [31 – 47]	40 [36 – 49]	40 [25 – 43]	0.411
***Secondary endpoints***				
*Over the first 24 hours following randomization*				
Moderate to severe dyspnea, *n (%)*	17 (77)	8 (73)	9 (82)	1.000
Average anxiety-VAS, *median (IQR)*	22 [10 – 45]	22 [10 – 33]	29 [10 – 62]	0.411
Moderate to severe anxiety*, n (%)*	9 (64)	4 (57)	5 (71)	1.000
Average respiratory rate, *per min, median (IQR)*	25 [23 – 30]	23 [21 – 26]	27 [24 – 30]	0.088
Average Glasgow coma scale, *median (IQR)*	15 [15 – 15]	15 [15 – 15]	15 [15 – 15]	0.488
Glasgow coma scale ≤12, *n (%)*	2 (9)	0 (0)	2 (18)	0.476
Sleep quality-VAS, *median (IQR)*	30 [10 ; 50]	44 [15 ; 50]	15 [4 ; 45]	0.432
Nurses satisfaction with protocol-VAS, *median (IQR)*	7 [7 – 8]	8 [8 – 9]	7 [5 – 7]	0.011
*Over the first 48 hours following randomization*				
Average nausea-VAS, *median (IQR)*	0 [0 – 20]	0 [0 – 0]	11 [0 – 21]	0.151
Average constipation-VAS, *median (IQR)*	0 [0 – 32]	2 [0 – 38]	0 [0 – 23]	0.772

Quantitative variables are expressed as median (interquartile range [IQR]) and qualitative variables are expressed as frequency (percentage)

*IQR* interquartile range, *VAS* visual analog scale

### Secondary outcomes

**Fig. 3:**
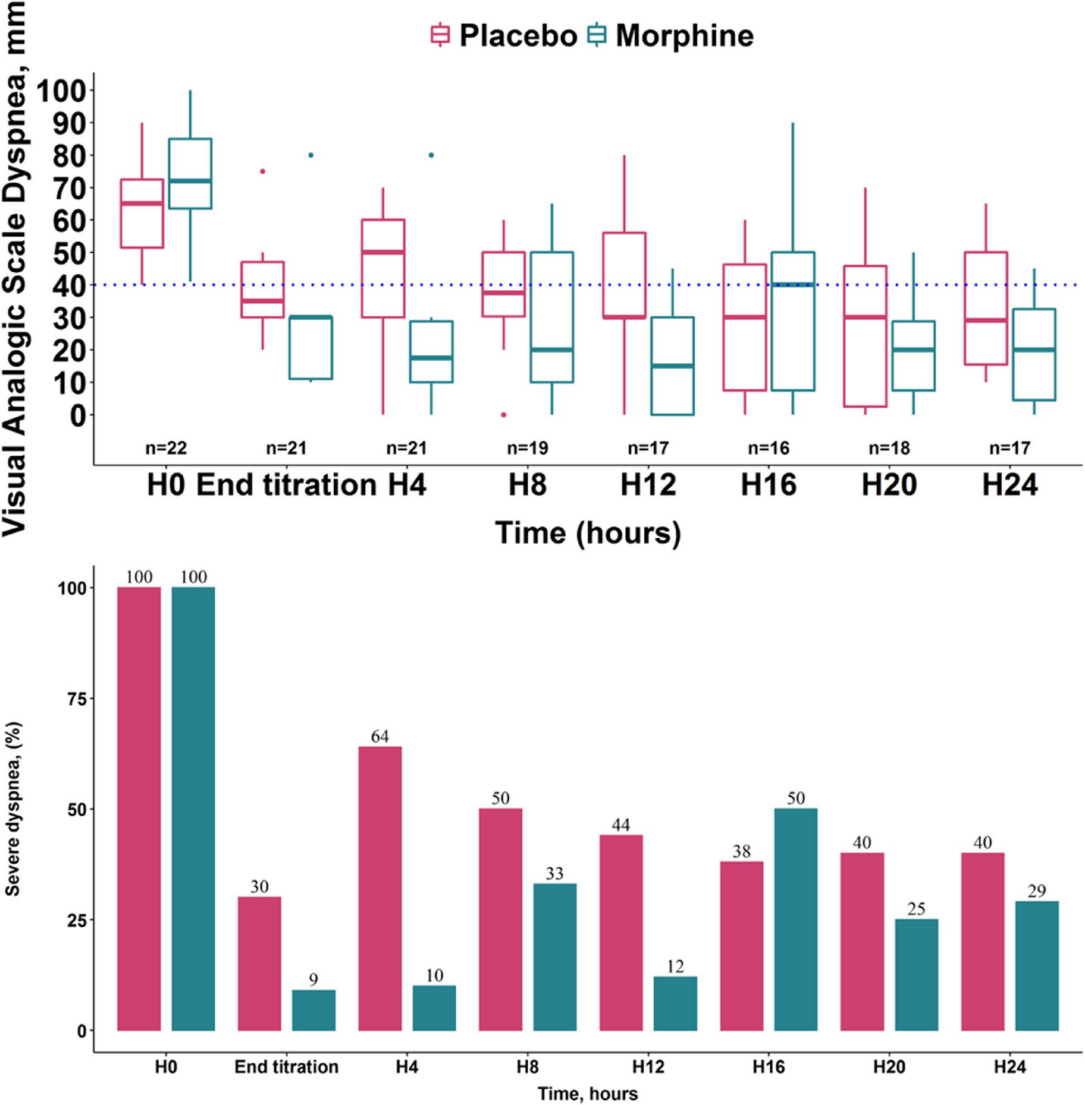
Visual analog scale for dyspnea (upper panel) and prevalence of moderate to severe dyspnea (lower panel) in the Morphine and the Placebo group on enrolment, at the end of intravenous titration and every four hours during the 24 hours following randomization

Figure 3 shows dyspnea-VAS and the proportion of patients exhibiting moderate to severe dyspnea at baseline, at the end of intravenous titration and every 4 hours during the 24 hours of follow-up. Dyspnea-VAS was lower in the Morphine group than in the Placebo group at the end of intravenous titration (30 [11 – 30] ml vs. 35 [30 – 44] ml, *p*=0.044) and four hours later (18 [10 – 29] ml vs. 50 [30 – 60] ml, *p*=0.043). There was no significant difference between the two groups at other time points in terms of dyspnea-VAS. At the end of intravenous titration, the proportion of patients exhibiting moderate to severe dyspnea was lower in the Morphine group than in the Placebo group (9% vs 30%, *p*< 0.001). There was no difference between the two groups at other time points in terms of proportion of patients exhibiting moderate to severe dyspnea.

Table 2 shows main secondary outcomes over the 24-hour study period (see also Table E1 in the Online Supplement for all other planned secondary outcomes). The average respiratory rate over the 24 hours was higher in the Morphine group than in the Placebo group. There was no significant difference between the two groups in the incidence of moderate to severe dyspnea or anxiety and in terms of average anxiety-VAS and Glasgow coma scale over the 24 hours, proportion of patients with a Glasgow coma scale ≤12, constipation and nausea. Nurses’ satisfaction with the protocol was higher in the Placebo group than in the Morphine group (8 [8, 9] mm vs 7 [5–7] mm *p*= 0.011).

### Adverse events

Table 3 shows adverse events over the 48 hours following randomization. The incidence of delirium, Glasgow coma scale ≤9, and severe pruritus, nausea and constipation was not significantly different between the two groups. In the Morphine group, 45% (*n*=5) of patients were intubated vs. 9% (*n*=1) in the Placebo group (*p*=0.149).

**Table 3:** Adverse events over the 48 hours following randomization

**Adverse events**	**All patients** **(*n*=22)**	**Placebo** **(*n*=11)**	**Morphine** **(*n*=11)**	***P* value**
**Adverse events regardless of severity, *n (%)***	9 (41)	2 (18)	7 (64)	0.080
Pruritus grade ≥4, *n (%)*	0 (0)	0 (0)	0 (0)	1.000
Nausea grade ≥3, *n (%)*	0 (0)	0 (0)	0 (0)	1.000
Constipation grade ≥4, *n (%)*	0 (0)	0 (0)	0 (0)	1.000
Bradypnea <12 cycles per minute, *n (%)*	0 (0)	0 (0)	0 (0)	1.000
Adverse events leading to stop treatment, *n (%)*	4 (18%)	1 (9)	3 (27%)	0.586
**Serious adverse events, *n (%)***	7 (32)	1 (9)	6 (55)	0.063
Glasgow coma scale<8, *n (%)*	1 (5)	0	1 (9)	1.000
Intubation, *n (%)*	6 (27)	1 (9)	5 (45)	0.149
Worsening of respiratory failure requiring intubation, *n (%)*	5 (23)	1 (9)	4 (36)	0.311
Death, *n (%)*	4 (18)	1 (9)	3 (27)	0.586

Qualitative variables are expressed as frequency (percentage)

**Fig. 4:**
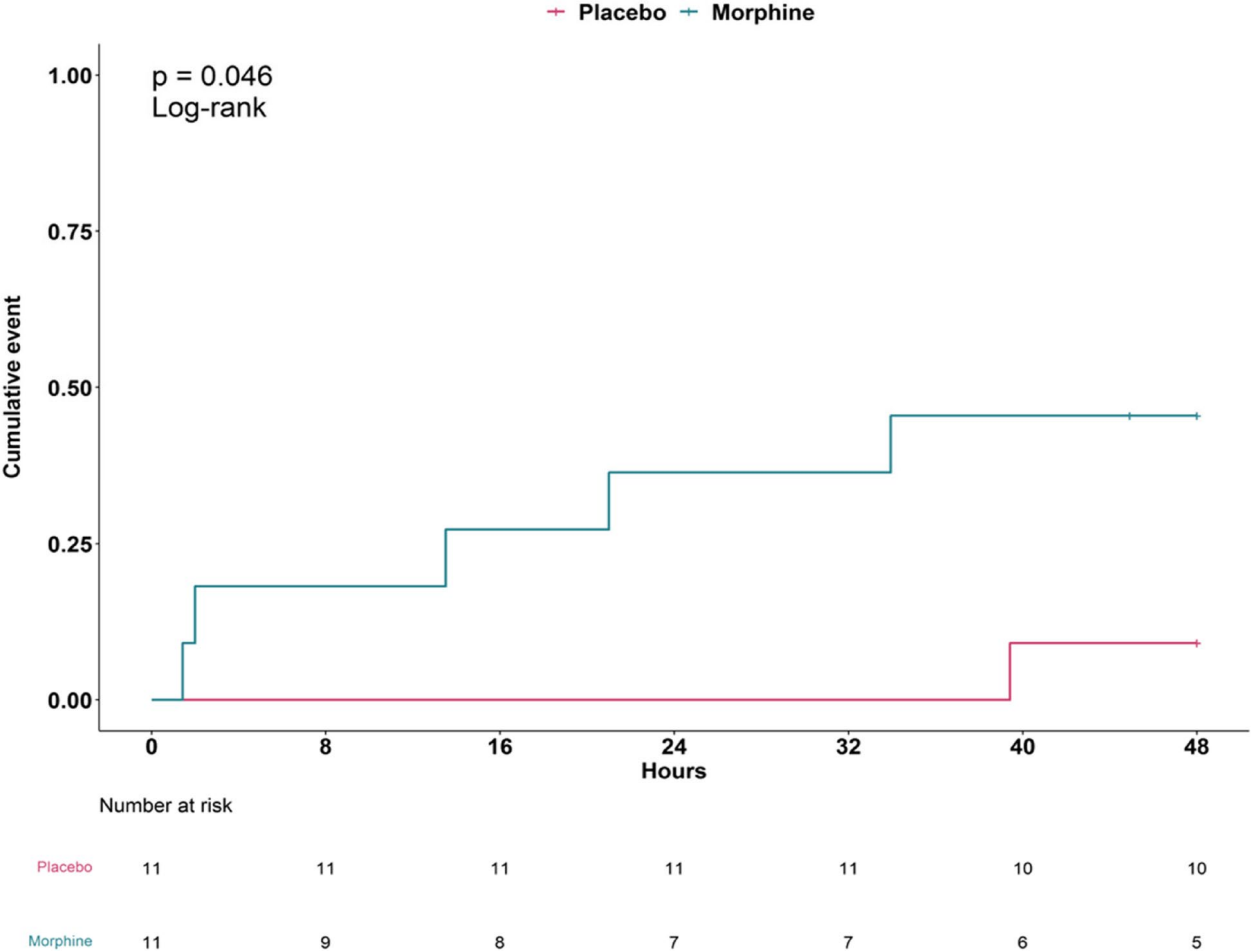
Cumulative risk for intubation over the 48 hours following randomization in the Morphine and in the Placebo group

The cumulative probability of intubation was higher in the Morphine group than in the Placebo group (Log rank, *p*=0.046, Fig. 4).

Three patients died in the Morphine group and one in the Placebo group (*p*=0.586).

## 42Discussion

In patients admitted to the ICU for ARF, this phase 2 pilot randomized controlled trial found no significant benefit of low-dose morphine on average dyspnea over 24 hours, although intravenous morphine titration significantly reduced dyspnea during the first four hours. Of concern was the fact that morphine was associated with an increased risk of intubation.

In recent years, dyspnea has become a matter of concern in ICU patients [7]. Indeed, dyspnea is frequent and severe in patients admitted for ARF [3, 31]. Dyspnea is associated with anxiety [3, 5, 32]. It is also associated with a higher prevalence of post-traumatic stress disorders. Relieving dyspnea should be a major target, such as controlling pain. When significant dyspnea persists despite treating the cause of ARF and administering a non-invasive respiratory support, it is permissible to consider the administration of opioids, in the absence of any other pharmacological approach, and based on their known effect on dyspnea in other clinical contexts. Original research and subsequent meta-analyses have shown that morphine successfully relieves dyspnea in patients with terminal cancer [13], cardiac failure [14], idiopathic pulmonary fibrosis [16] and chronic obstructive pulmonary disease [20, 25] In the ICU, recent data suggest that morphine successfully relives dyspnea in intubated patients [33], but no randomized trial had been conducted in ICU patients exhibiting moderate to severe dyspnea upon admission. This is the reason why we decided to conduct the present study. Morphine did improve dyspnea transiently, but failed to show any significant effect on the 24-hour average dyspnea as we defined it. One of the potential explanations is that the subcutaneous dosage of the repeated administrations was not high enough. Another explanation is that in both groups, patients received etiological and symptomatic treatment of dyspnea with bronchodilators, hydro-sodium depletion, anti-infectious therapies and ventilatory support with NIV, high-flow oxygen through nasal cannula or standard oxygen therapy. These therapies have a known effect in relieving dyspnea which, although inconstant, can lead to a floor effect, with dyspnea decreasing more rapidly in the Morphine group [34]. For instance, from the 8^th^ hour after inclusion, dyspnea-VAS was less than 40 mm in both groups, with prevalence of severe dyspnea that was less than 50%. With this in mind, we acknowledge that the choice of the 24-hour average dyspnea as the primary outcome of this study might have been a mistake: to draw a crude analogy, morphine is expected to be effective before fracture reduction, and much less so 24 hours afterwards. Therefore, regarding patients' comfort, the "end-of-titration" and "four-hour" dyspnea outcomes might be more relevant than the 24-hour average outcome. Another hypothesis would be that there was too much time between dyspnea ratings, possibly combined with too low a dose of morphine, which meant dyspnea was already increasing again at the time of the rating. It is also interesting to note that there is a wide dispersion (visible in Fig. 3), possibly due to significant variations in volume of distribution or pharmacokinetic effects, since morphine worked for the first hour, so the subcutaneous form dispensed afterwards may not be the most suitable.

We were struck by the magnitude of the effect observed in the placebo arm of the study. This efficacy had already been found in other studies looking at the relief of dyspnea, one of which failed to demonstrate the efficacy of sertraline in relieving chronic dyspnea [35] and the other the inability of nefopam to relieve experimental dyspnea in healthy volunteers [36]. In these studies, the placebo effect could modify the anticipation processes recognized as determinants of the experience of dyspnea [37]. Another mechanism involved could be the Hawthorne effect: participation in a clinical trial focusing on dyspnea would be sufficient to generate clinical benefits, by enabling patients to realize that their condition is being observed and is therefore no longer ignored [38]. It is therefore not surprising that participation in a clinical trial focusing on dyspnea should be sufficient to generate clinical benefits.

Of major concern was the higher proportion of patients intubated in the Morphine group. Beyond the efficiency of opioids in relieving dyspnea, several studies have pointed to their safety in patients with respiratory disorders, which is why guidelines from the American College of Chest Physicians [26], the Canadian Thoracic Society [39] and the American Thoracic Society [40] advocate the use of opioids for persistent dyspnea. Although opioids are known to depress respiratory drive, most studies conducted in dyspneic patients without ARF have shown that their use was not associated with a significant decrease in respiratory rate and pulsed oxygen saturation or an increase in PaCO_2_ [16, 18, 21, 41, 42]. Unfortunately, our observations do not go in this direction. Although morphine was not associated with a significant decrease in the level of consciousness (assessed by the Glasgow Coma scale), we observed a higher incidence of intubation in the Morphine group than in the Placebo group, noting that two of the patients intubated in the morphine group were intubated during the titration period. This strongly tempers the idea that could derive from our results that morphine could be useful not over 24 hours but at the very initial phase of ARF: finding the amount of opioids that may relieve dyspnea without worsening ARF and precipitating intubation might well be impossible. Of notice, patients in the Morphine group were more likely to have a chronic respiratory disease and had a higher baseline respiratory rate, which may suggest that they were more severe and hence may explain why the intubation rate was higher in the Morphine group. Finally, our study raised the potential interest of non-pharmacologic interventions such as sensory interventions targeting the brain rather than the respiratory system. The principle of these interventions is to modulate the emotional and affective component of dyspnea. Recent data in mechanically ventilated patients experiencing dyspnea show that exposure to relaxing music and exposure to facial air flux delivered by a fan significantly decrease dyspnea [43]. These interventions have no toxicity.

The strength of our study is to be the first randomized controlled trial to evaluate the potential benefit of opioids on dyspnea in patients admitted to the ICU with ARF. We used a double-blind design to limit bias, in particular classification bias, with a primary outcome that could suffer from subjective assessment. This study has several major limitations. First, due to the small sample size, the study is clearly underpowered. We designed it as a pilot phase 2 study and therefore our results should be considered as exploratory. We calculated the sample size based on the benefit of morphine in non-critically ill patients. We acknowledge that the small sample size may limit the capacity to account for variables such as underlying diseases, concomitant therapies, or patient anxiety levels. However, the aim of randomization is to balance characteristics between groups. In addition, the exclusion rate was high due to very stringent non-inclusion criteria, with the purpose of enrolling patients corresponding as much as possible to our target population, which we found crucial for a pilot phase 2 trial. Second, although we showed an increased incidence of intubation in the Morphine group, it is important to keep in mind that there were no predefined criteria for intubation. Third, the switch from intravenous titration to subcutaneous administration seemed to be associated with a relapse of dyspnea. Intravenous patient-controlled analgesia might be a promising alternative to subcutaneous administration.

## Conclusion

In conclusion, this single-center phase 2 pilot randomized controlled trial not only failed to show a benefit of morphine in relieving dyspnea over 24 hours in patients with ARF and severe dyspnea admitted to the ICU trial, but also showed that morphine was associated with a higher intubation rate. Because dyspnea is a major issue in critically ill patients, future studies should search for a protocol of opioid administration that relieves dyspnea without worsening ARF severity.

### Supplementary Information

Supplementary Material 1.

## Data Availability

No datasets were generated or analysed during the current study.
