# Supplementary material for: Low dose of morphine to relieve dyspnea in acute respiratory failure: the OpiDys double-blind randomized controlled trial
Source: Respir Res. 2024 Jul 16;25:280. doi: 10.1186/s12931-024-02867-2 (PMC11251226; doi:10.1186/s12931-024-02867-2)
Supplement: Supplementary file 1 — Supplementary Material 1. [file 12931_2024_2867_MOESM1_ESM.docx]

**Low dose of morphine to relieve dyspnea in acute respiratory failure: the OpiDys double-blind randomized controlled trial**

**Online Supplement**

**Table E1. Secondary outcomes**

**Table E1. Secondary outcomes**

| **Endpoints** | **All patients (n = 22)** | **Placebo (n = 11)** | **Morphine (n = 11)** | **P value** |
| --- | --- | --- | --- | --- |
| ***Secondary endpoints*** | | | | |
| *Over the first 24 hours following randomization* | | | | |
| Mean anxiety-VAS, *median (IQR)* | 22 [10 – 45] | 22 [10 – 33] | 29 [10 – 62] | 0.411 |
| Severe anxiety, *n (%)* | 9 (64) | 4 (57) | 5 (71) | 1.000 |
| Mean respiratory rate, min-1, *median (IQR)* | 25 [23 – 30] | 23 [21 – 26] | 27 [24 – 30] | 0.088 |
| Mean Glasgow coma scale, *median (IQR)* | 15 [15 – 15] | 15 [15 – 15] | 15 [15 – 15] | 0.488 |
| Glasgow Coma Scale ≤ 12, *n (%)* | 2 (9) | 0 (0) | 2 (18) | 0.476 |
| Eye dryness-VAS, *median (IQR)* | 0 [0 – 5] | 0 [0 – 0] | 5 [0 – 20] | 0.137 |
| Nose dryness-VAS, *median (IQR)* | 10 [0 – 40] | 5 [0 – 36] | 10 [0 – 35] | 0.756 |
| Feeling of gastric distension-VAS, *median (IQR)* | 0 [0 – 0] | 0 [0 – 0] | 0 [0 – 4] | 0.600 |
| Sleep quality-VAS, median (IQR) | 30 [10 – 50] | 44 [15 – 50] | 15 [4 – 45] | 0.432 |
| Sleep duration, hours, median (IQR) | 4 [4 – 6] | 4 [2 – 5] | 5 [4 – 6] | 0.188 |
| Duration of oxygen, hours, *median (IQR)* | 16 [8 – 24] | 16 [9 – 24] | 19 [10 – 23] | 0.631 |
| Oxygen tolerance-VAS, *median (IQR)* | 6 [0 – 25] | 11 [6 – 36] | 0 [0 – 15] | 0.487 |
| Number of NIV sessions, *median (IQR)* | 1 [1 – 2] | 1 [1 – 2] | 2 [1 – 2] | 0.696 |
| Duration of NIV, hours *median (IQR)* | 8 [8 – 14] | 8 [8 – 8] | 16 [12 – 20] | 0.285 |
| NIV tolerance-VAS, *median (IQR)* | 38 [35 – 50] | 49 [44 – 54] | 35 [18 – 42] | 0.400 |
| Duration of HFNO, hours, *median (IQR)* | 19 [15 – 22] | 21 [16 – 24] | 18 [12 – 19] | 0.294 |
| HFNO tolerance-VAS, *median (IQR)* | 20 [3 – 70] | 13 [4 – 38] | 50 [25 – 75] | 0.593 |
| Change of non-invasive respiratory support, *n (%)* | 12 (55) | 5 (45) | 7 (64) | 0.670 |
| Nurses satisfaction with protocol-VAS, *median (IQR)* | 7 [7 – 8] | 8 [8 – 9] | 7 [5 – 7] | 0.011 |
| *Over the first 48 hours following randomization* | | | | |
| Nausea-VAS, *median (IQR)* | 0 [0 – 20] | 0 [0 – 0] | 11 [0 – 21] | 0.151 |
| Constipation-VAS, *median (IQR)* | 0 [0 – 32] | 2 [0 – 38] | 0 [0 – 23] | 0.772 |

Quantitative variables are expressed as median (interquartile range [IQR]) and qualitative variables are expressed as frequency (percentage).

IQR, interquartile range; VAS, visual analog scale; NIV, non-invasive ventilation; HFNO, high flow nasal oxygen.
